# Negative differential conductance and super-Poissonian shot noise in single-molecule magnet junctions

**DOI:** 10.1038/srep08730

**Published:** 2015-03-04

**Authors:** Hai-Bin Xue, Jiu-Qing Liang, Wu-Ming Liu

**Affiliations:** 1 College of Physics and Optoelectronics, Taiyuan University of Technology, Taiyuan 030024, China; 2 Institute of Theoretical Physics, Shanxi University, Taiyuan 030006, China; 3 Beijing National Laboratory for Condensed Matter Physics, Institute of Physics, Chinese Academy of Sciences, Beijing 100190, China

## Abstract

Molecular spintroinic device based on a single-molecule magnet is one of the ultimate goals of semiconductor nanofabrication technologies. It is thus necessary to understand the electron transport properties of a single-molecule magnet junction. Here we study the negative differential conductance and super-Poissonian shot noise properties of electron transport through a single-molecule magnet weakly coupled to two electrodes with either one or both of them being ferromagnetic. We predict that the negative differential conductance and super-Poissonian shot noise, which can be tuned by a gate voltage, depend sensitively on the spin polarization of the source and drain electrodes. In particular, the shot noise in the negative differential conductance region can be enhanced or decreased originating from the different formation mechanisms of negative differential conductance. The effective competition between fast and slow transport channels is responsible for the observed negative differential conductance and super-Poissonian shot noise. In addition, we further discuss the skewness and kurtosis properties of transport current in the super-Poissonian shot noise regions. Our findings suggest a tunable negative differential conductance molecular device, and the predicted properties of high-order current cumulants are very interesting for a better understanding of electron transport through single-molecule magnet junctions.

Electronic transport through a single-molecule magnet (SMM) has been intensively studied both experimentally^1^,^2^,^3^,^4^,^5^,^6^,^7^,^8^,^9^,^10^,^11^ and theoretically^12^,^13^,^14^,^15^,^16^,^17^,^18^,^19^,^20^,^21^,^22^,^23^,^24^,^25^,^26^ due to its applications in molecular spintronics^27^, but these investigations were focused mainly on the differential conductance or average current. Although the shot noise of electron transport through a SMM has not yet been observed experimentally, new techniques based on carbon nanotubes have been proposed for its possible realization^28^. Recently, the current noise properties of electron transport through a SMM have been attracting much theoretical research interests^29^,^30^,^31^,^32^,^33^,^34^,^35^,^36^ due to they can provide a deeper insight into the nature of transport mechanisms that cannot be obtained by measuring the differential conductance or average current^37^,^38^. For example, the super-Poissonian shot noise can be used to reveal the information about the internal level structure of the SMM, the left-right asymmetry of the SMM-electrode coupling^32^,^33^, and the angle between the applied magnetic field and the SMM's easy axis^34^; and distinguish the two types of different nonequilibrium dynamics mechanisms, namely, the quantum tunneling of magnetization process and the thermally excited spin relaxation^35^. In particular, the frequency-resolved shot noise spectrum of artificial SMM, e.g., a CdTe quantum dot doped with a single *S* = 5/2 Mn spin, can allow one to separately extract the hole and Mn spin relaxation times via the Dicke effect^36^.

Among these observed or predicted characteristics, the negative differential conductance (NDC) is especially concerned due to the SMM's potential applications in a new generation of molecule-based memory devices and logic circuits. On the other hand, the shot noise is usually the sub-Poissonian statistics in non-interacting fermion systems originating from the Pauli exclusion principle. Thus, the super-Poissonian shot noise is another important characteristic of transport current. Here, the so-called Fano factor, which is used to characterize the shot noise and defined as the ratio of zero-frequency shot noise and the full Poisson noise, is smaller than one for sub-Poissonian shot noise and exceeds one for super-Poissonian shot noise. According to the definition of Fano factor, the super-Poissonian shot noise, namely, the super-Poissonian distribution of electron counts has a width that is broader than its mean, whereas for a Poissonian distribution the width and the mean have the same value. For the SMM weakly coupled to two normal metal electrodes, the NDC formation mechanism originates essentially from the non-equilibrium electron occupation of the system eigenstates entering bias voltage window^14^,^32^,^39^, namely, the increased current magnitudes of the new opened transport channels do not compensate the decreased current magnitude(s) of the already opened transport channel(s), and the shot noise in this NDC region is obviously enhanced even up to a super-Poissonian shot noise value. In particular, the occurrence of super-Poissonian shot noise depends on the effective competition between different transport channels, thus, the SMM's internal level structure and the left-right asymmetry of the SMM-electrode coupling, which can tune the SMM transport channels, have an important influence on the super-Poissonian shot noise properties^32^,^33^,^34^. Whereas for the SMM weakly coupled to two electrodes with either one or both of them being ferromagnetic, the spin polarization of the source and drain electrodes play an important role in the forming speed of the correlated SMM eigenstates involved in the electron tunneling processes, and thus have a remarkable influence on the transport channels entering bias voltage window^21^,^31^,^40^. Consequently, the spin polarization of the source and drain electrodes will have an significant impact on the NDC and super-Poissonian shot noise properties of this SMM system. However, the influences of the spin polarization of the source and drain electrodes on the NDC and super-Poissonian shot noise in the SMM system have not yet been revealed.

The goal of this report is thus to study the influences of the spin polarization of the source and drain electrodes and the applied gate voltage on the NDC and super-Poissonian shot noise in a SMM weakly coupled to two electrodes with either one or both of them being ferromagnetic, and discuss the underlying mechanisms of the observed NDC and super-Poissonian shot noise. It is demonstrated that the gate-voltage-controlled NDC and super-Poissonian shot noise depend sensitively on the spin polarization of the source and drain electrodes. In particular, whether the shot noise in the NDC region being enhanced or not is associated with the formation mechanism of the NDC. Moreover, the skewness and kurtosis in the super-Poissonian shot noise regions show the crossovers from a large positive (negative) to a large negative (positive) values, which also depend on the spin polarization of the source and drain electrodes. These observed characteristics are very interesting for a better understanding of electron transport through single-molecule magnet junctions and will allow for experimental tests in the near future.

## Results

### Single-molecule magnet junction

**Figure 1:**
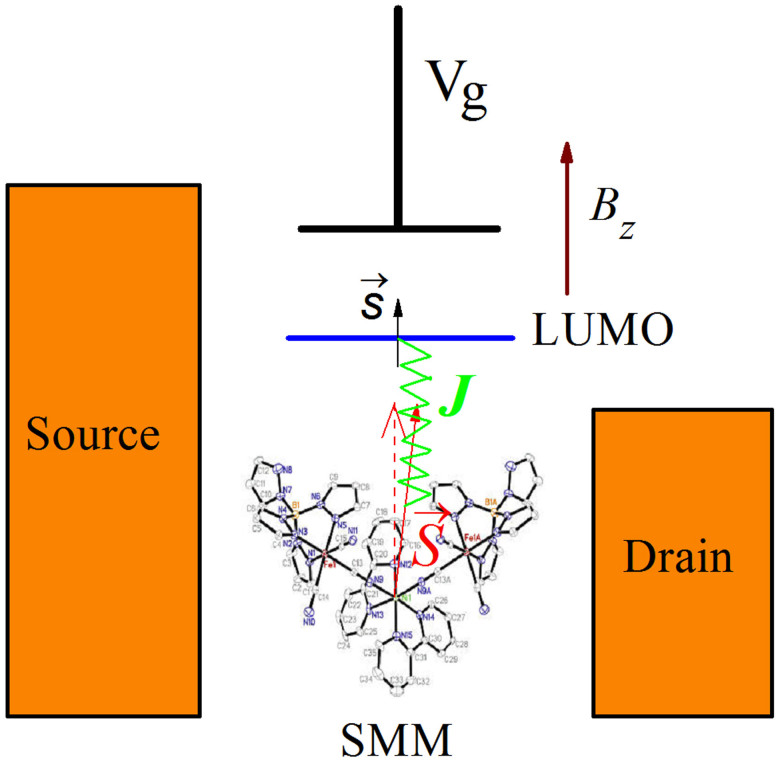
Schematic representation of a single-molecule magnet (SMM) weakly coupled to two leads. The SMM consists of the lowest unoccupied non-degenerate molecular orbital (LUMO), which can be tuned by a gate voltage *V_g_*, the phenomenological giant spin 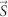, and the uniaxial anisotropy energy *K*_2_(*S_z_*)^2^. The exchange coupling between the conduction electron spin 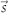 in the LUMO and the SMM spin 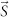 is denoted by *J*. The external magnetic field *B_z_* is applied along the easy axis of the SMM. Here, we consider three different electrode configurations, namely, (i) the ferromagnetic lead (Source) - SMM - normal-metal lead (Drain), (ii) the normal-metal lead (Source) - SMM - ferromagnetic lead (Drain), (iii) the ferromagnetic lead (Source) - SMM - ferromagnetic lead (Drain).

The SMM junction consists of a SMM weakly coupled to two electrodes, see Fig. 1. The SMM is characterized by the lowest unoccupied non-degenerate molecular orbital (LUMO), the phenomenological giant spin 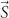, and the uniaxial anisotropy. The SMM Hamiltonian is thus described by
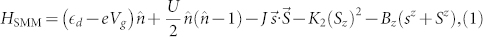
Here, the first two terms depict the LUMO, 
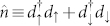
 and *U* are respectively the electron number operator and the Coulomb repulsion between two electrons in the LUMO, with 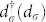 being the electron creation (annihilation) operators with spin *σ* and energy *ε_d_* (which can be tuned by a gate voltage *V_g_*) in the LUMO. The third term describes the exchange coupling between the conduction electron spin 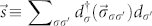 in the LUMO and the SMM spin 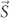, with 
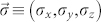
 being the vector of Pauli matrices. The forth term stands for the anisotropy energy of the SMM whose easy-axis is *Z*-axis (*K*_2_ > 0). The last term denotes Zeeman splitting. For simplicity, we assume an external magnetic field 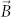 is applied along the easy axis of the SMM.

The relaxation in the two electrodes is assumed to be sufficiently fast so that their electron distributions can be described by equilibrium Fermi functions. The two electrodes are thus modeled as noninteracting Fermi gases and the corresponding Hamiltonians read
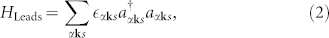
where 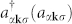 is the electron creation (annihilation) operators with energy *ε_α_*_**k***σ*_, momentum **k** and spin *s* in *α* (*α* = *L*, *R*) electrode, and the index *s* = + (−) denotes the majority (minority) spin states with the density of states 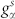. The electrode polarization is characterized by the orientation of the polarization vector **p***_α_* and its magnitude is defined as 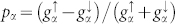. Here, the polarization vectors **p***_L_* (left electrode) and **p***_R_* (right electrode) are parallel to the spin quantization *Z* axis, and spin-up ↑ and spin-down ↓ are respectively defined to be the majority spin and minority spin of the ferromagnet. The tunneling between the SMM and the two electrodes are thus described by
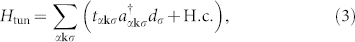
Here, for the ferromagnetic electrode case, the electronic tunneling rates depend on the conduction-electron spin, namely, 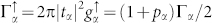 and 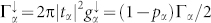, where the tunneling amplitudes *t_α_* and the density of the state 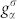 are assumed to be independent of wave vector and energy, and 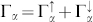; while for the normal-metal electrode case, *p_α_* = 0, thus, 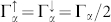.

In addition, we assume that the bias voltage is symmetrically entirely dropped at the SMM-electrode tunnel junctions, i.e., *μ_L_* = −*μ_R_* = *V_b_*/2, which implies that the levels of the SMM are independent of the applied bias voltage, and choose meV as the unit of energy. In the Coulomb blockade regime, the occurrence or absence of super-Poissonian shot noise is related to the sequential tunneling gap 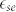 that being the energy difference between the ground state of charge *N* and the first excited state of charge *N* − 1, and the “vertical” energy gap 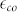 between the ground state of charge *N* and the first excited state of the same charge^41^. In the present work, we only study the electron transport above the sequential tunneling threshold, namely, 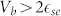. In this bias voltage region, the conduction electrons have sufficient energy to overcome the Coulomb blockade and tunnel sequentially through the SMM. It should be noted that the transport current in the Coulomb blockade regime is exponentially suppressed and the co-tunneling tunneling process is dominant in the electron transport, thus, the normalized shot noise will deviate from the present results when taking co-tunneling into account. In order to better discuss the occurrence mechanisms of the NDC and the super-Poissonian shot noise, the spin of the SMM (e.g., the cyanide-bridged trinuclear 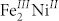 SMM with an *S* = 2 ground states^42^) is chosen as *S* = 2. The other parameters of the SMM are chosen as: *ε_d_* = 0.2, *U* = 0.1, *J* = 0.1, *K*_2_ = 0.04, *B* = 0.08, Γ*_L_* = Γ*_R_* = Γ = 0.002 and *k_B_T* = 0.02.

We first study numerically the effects of the spin polarization of the two electrodes and the applied gate voltage on the NDC and super-Poissonian shot noise in the three different electrode configurations (see Fig. 1), namely, (i) the ferromagnetic lead (Source) - SMM - normal-metal lead (Drain) (i.e., the F-SMM-N system), (ii) the normal-metal lead (Source) - SMM - ferromagnetic lead (Drain) (i.e., the N-SMM-F system), (iii) the ferromagnetic lead (Source) - SMM - ferromagnetic lead (Drain) (i.e., the F-SMM-F system).

### The ferromagnetic lead (Source) - SMM - normal-metal lead (Drain)

For the F-SMM-N system considered here, the conduction electron will tunnel into the SMM from the ferromagnetic lead and then tunnel out of the SMM onto the normal-metal lead. The strengths of tunneling coupling of the SMM with two electrodes can be expressed as 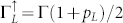, 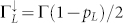 and 
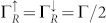
. Since only the energy eigenvalues of singly-occupied and doubly-occupied eigenstates 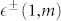 and 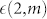 depend on the gate voltage *V_g_*, the transition between the singly- and doubly-occupied eigenstates, or between the empty- and singly-occupied eigenstates first entering bias voltage window can be tuned by the gate voltage^14^. For example, for a relatively small or negative gate voltage, the transition from the singly- to empty-occupied eigenstates first takes place; while for a large enough gate voltage that from the double- to singly-occupied eigenstates first occurs.

**Figure 2:**
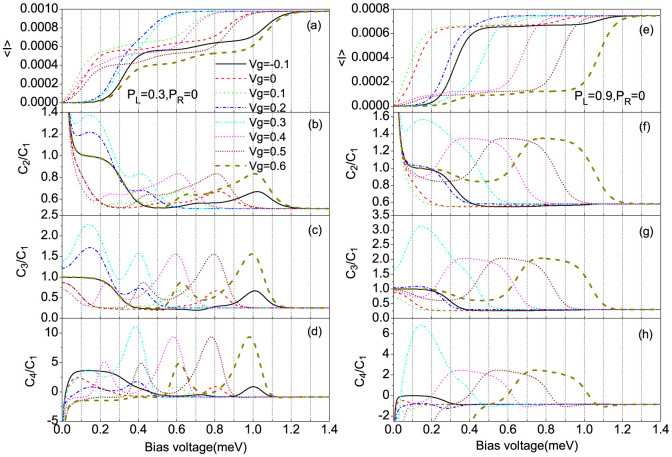
The average current (〈*I*〉), shot noise (*C*_2_/*C*_1_), skewness (*C*_3_/*C*_1_) and kurtosis (*C*_4_/*C*_1_) vs bias voltage for different gate voltages, here *C_k_* is the zero-frequency *k*-order cumulant of current fluctuations. (a), (b), (c) and (d) for *p_L_* = 0.3 and *p_R_* = 0; (e), (f), (g) and (h) for *p_L_* = 0.9, *p_R_* = 0. The SMM parameters: *S* = 2, *ε_d_* = 0.2, *U* = 0.1, *J* = 0.1, *K*_2_ = 0.04, *B* = 0.08, Γ*_L_* = Γ*_R_* = Γ = 0.002 and *k_B_T* = 0.02.

Figures 2(a) and 2(b), 2(e) and 2(f) show the average current and shot noise as a function of the bias voltage for different gate voltages *V_g_* with *p_L_* = 0.3 and *p_L_* = 0.9. For a large enough spin polarization of source electrode *p_L_*, the super-Poissonian shot noise is observed when the transition from the doubly- and singly-occupied eigenstates first participates in the electron transport with the bias voltage increasing, see the short dashed, short dash-dotted and thick dashed lines in Fig. 2(f), whereas for the QD system the super-Poissonian noise dose not appear^43^. This characteristic can be understood in terms of the effective competition between fast and slow transport channels^32^,^33^,^34^,^41^,^44^,^45^,^46^,^47^,^48^,^49^,^50^ and the forming speed of the new correlated eigenstates^49^. The current magnitudes of the SMM transport channels can be expressed as^14^,^32^,^34^
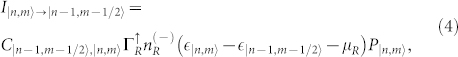

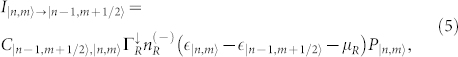
where *C*_|*n* − 1,*m* ± 1/2〉,|*n*,*m*〉_ = |〈*n* − 1, *m* ± 1/2|*d_σ_* |*n*, *m*〉|^2^ is a constant which related to the two SMM eigenstates but independent of the applied bias voltage, and *P*_|*n*,*m*〉_ is the occupation probability of the SMM eigenstate |*n*, *m*〉. Here, the Fermi function 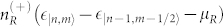 changes very slowly with increasing bias voltage above the sequential tunneling threshold, namely, 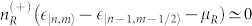, thus, 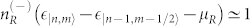. The current magnitude of the SMM transport channel is thus mainly determined by the occupation probability *P*_|*n*,*m*〉_ and 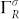.

**Figure 3:**
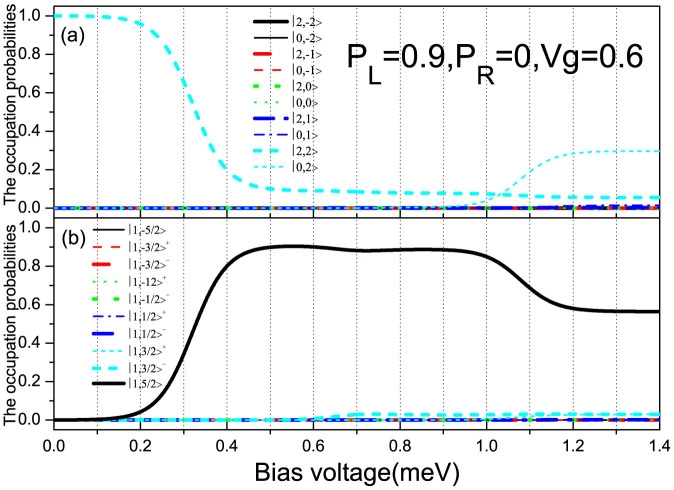
The occupation probabilities of the SMM eigenstates vs bias voltage for *p_L_* = 0.9, *p_R_* = 0 and *V_g_* = 0.6. The SMM parameters are the same as in Fig. 2.

In order to give a qualitative explanation for the underlying mechanism of the observed super-Poissonian shot noise, we plot the occupation probabilities of the SMM eigenstates as a function of bias voltage for *p_L_* = 0.9 and *V_g_* = 0.6 in Fig. 3. With increasing bias voltage, the transport channel 
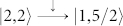
 begins to participate in the electron transport. When the bias voltage increases up to about 0.6 meV, the new transport channel 
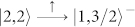
 enters the bias voltage window. In this situation, the conduction electron can tunnel out SMM via the two transport channels 
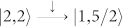
 and 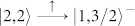. For the F-SMM-N system, the electron tunneling between the SMM and the drain electrode (normal-metal lead) is independent of the conduction electron spin, thus the tunneling process mainly relies on the forming speed of the new doubly-occupied eigenstate |2, 2〉. In the case of 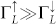, a new doubly-occupied eigenstate |2, 2〉 can be quickly formed when the spin-up electron tunnels out of the SMM; whereas for the case of the spin-down electron tunneling out of the SMM, the forming of the corresponding new doubly-occupied eigenstate |2, 2〉 takes a relatively longer time. Thus, for a large enough *p_L_*, the fast transport channel 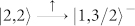 can be modulated by the correlated slow channel 
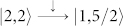
, which leads to the bunching effect of the conduction electrons being formed, and is responsible for the formation of the super-Poissonian noise. When *V_b_* > 0.9 meV, the transport channels 
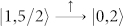
 and 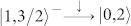 enter the bias volatge window, so that the two successive electron tunneling processes 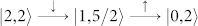 and 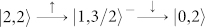 can be formed. Consequently, the formed active competition between the fast-and-slow transport channels is suppressed even destroyed with the current magnitudes of the two new transport channels increasing, which leads to the super-Poissonian shot noise being decreased and even to the sub-Poissonian.

### The normal-metal lead (Source) - SMM - ferromagnetic lead (Drain)

**Figure 4:**
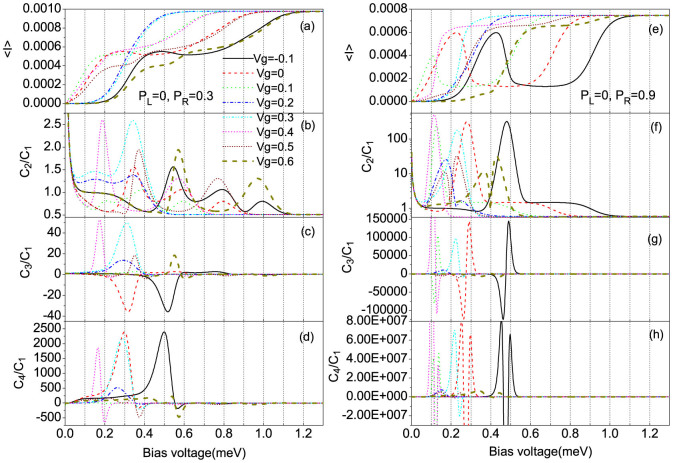
The average current (〈*I*〉), shot noise (*C*_2_/*C*_1_), skewness (*C*_3_/*C*_1_) and kurtosis (*C*_4_/*C*_1_) vs bias voltage for different gate voltages, here *C_k_* is the zero-frequency *k*-order cumulant of current fluctuations. (a), (b), (c) and (d) for *p_L_* = 0 and *p_R_* = 0.3; (e), (f), (g) and (h) for *p_L_* = 0, *p_R_* = 0.9. The SMM parameters are the same as in Fig. 2.

In the N-SMM-F system, the strengths of tunnel coupling between the SMM and the two electrodes are described by 
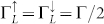
, 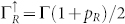, 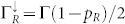. It is demonstrated that the NDC is observed for a small enough or negative gate voltage, and a relatively large spin polarization of drain electrode *p_R_*, see the solid and dashed lines in Figs. 4(a) and 4(e), especially for a large enough spin polarization *p_R_* a strong NDC takes place, see the solid and dashed lines in Fig. 4(e). Moreover, the shot noise can be significantly enhanced and reaches up to a super-Poissonian value when the magnitude of the total current begins to decrease, but the super-Poissonian value in the NDC region is then decreased with further increasing the bias voltage, see the solid and dashed lines in Figs. 4(b) and 4(f). While for a large enough gate voltage, the peaks of super-Poissonian shot noise are observed for a relatively large spin polarization *p_R_*, see the short dash-dotted and thick dashed lines in Figs. 4(b) and 4(f). The observed NDC and super-Poissonian shot noise characteristics can also be attributed to the mechanism of the fast-and-slow transport channels. Here, we take the *V_g_* = −0.1 and *V_g_* = 0.6 cases with *p_R_* = 0.9 as examples to illustrate these characteristics.

**Figure 5:**
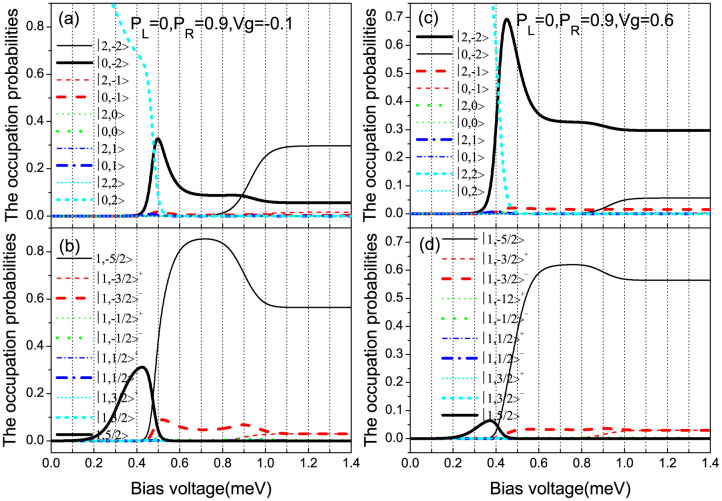
The occupation probabilities of the SMM eigenstates vs bias voltage for different gate voltages with *p_L_* = 0 and *p_R_* = 0.9. (a) and (b) for *V_g_* = −0.1; (c) and (d) for *V_g_* = 0.6. The SMM parameters are the same as in Fig. 2.

For the *V_g_* = −0.1 case, the transition from singly-occupied to empty eigenstates 
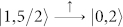
 first participates in the electron transport with increasing the bias voltage, see Figs. 5(a) and 5(b). When the bias voltage is larger than 0.33 meV, the SMM has a small probability of forming the empty-occupied eigenstate |0, −2〉, see the thick solid line in Fig. 5(a). If the spin-down electron tunnels into the SMM, the singly-occupied eigenstate |1, −5/2〉 can be formed. In this case, for a large enough spin polarization *p_R_*, namely, 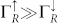, the spin-down electron will remain for a relatively long time in the SMM, so that the electron tunneling processes via the fast transport channels 
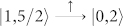
 and 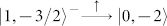 can be blocked and the conduction electrons appear the bunching effect. On the other hand, the current magnitude of the formed fast transport channel 
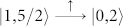
 begins to decrease with increasing the bias voltage up to about 4.25 meV, while that of the two new opened transport channels 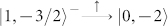 and 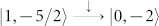 increase. Since the occupation probabilities of the eigenstates |1, −3/2〉^−^ and |1, −5/2〉, 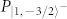 and *P*_|1,−5/2〉_ are much smaller that *P*_|1,5/2〉_, see Fig. 5(b), thus, for the 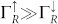 case the decreased current magnitude of transport channel 
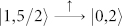
 is much larger than the increased current magnitudes of transport channels 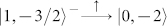 and 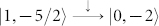. Thus, a strong NDC is observed, see the solid line in Fig. 4(e). Moreover, the active competition between the fast channel of current decreasing and the slow channels of current increasing can also obviously enhance the shot noise. Consequently, the shot noise is significantly enhanced by the above two mechanisms and reaches up to a very large value of super-Poissonian shot noise before the occupation probabilities 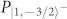 and *P*_|1,−5/2〉_ are larger than *P*_|1,5/2〉_, and *P*_|0,−2〉_ is larger than *P*_|0,2〉_. With the bias voltage further increasing, the value of super-Poissonian shot noise is decreased quickly but still remains the super-Poissonian distribution. This originates from the fact that the transport channels 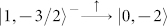 and 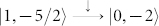 can form a new effective competition between the fast and slow transport channels. When the occupation probability *P*_|2,2〉_ is larger than *P*_|0,2〉_ (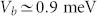), the active competition between the transport channels 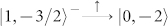 and 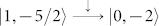 is destroyed by the new transport channel 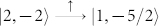 due to the electron tunneling process via the transport channel 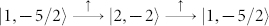 can occur, which is responsible for the super-Poissonian shot noise being decreased to the sub-Poissonian distribution.

As for the *V_g_* = 0.6 case, the transport channel 
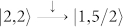
, which is a slow transport channel for the 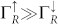 case, first participates in the electron transport, see Figs. 5(c) and 5(d). With the bias voltage increasing up to about 0.4 meV, the fast electron tunneling process via the transport channel 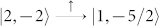 takes place, thus, the effective competition between fast and slow transport channels can form, and the shot noise is rapidly enhanced and reaches up to a relatively large super-Poissonian value. However, the new transport channels 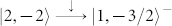 and 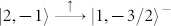 can be quickly opened with the bias voltage further increasing, then the fast transport channel 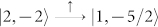 will be weakened when a spin-down electron tunnels out the SMM through the transition from the eigenstates |2, −2〉 to |1, −3/2〉^−^, so that the formed effective competition between fast and slow transport channels is suppressed and even destroyed. Moreover, when the transport channel 
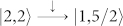
 does not participate in the quantum transport originating from the occupation probabilities *P*_|2,2〉_ and *P*_|1,5/2〉_ being approaching zero, the two transport channels 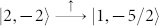 and 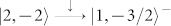 can not form a new effective competition between fast and slow transport channels due to a relatively fast electron tunneling process via 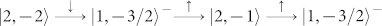 can take place. Consequently, the super-Poissonian shot noise is decreased quickly to a sub-Poissonian value and displays a sharp peak.

### The ferromagnetic lead (Source) - SMM - ferromagnetic lead (Drain)

**Figure 6:**
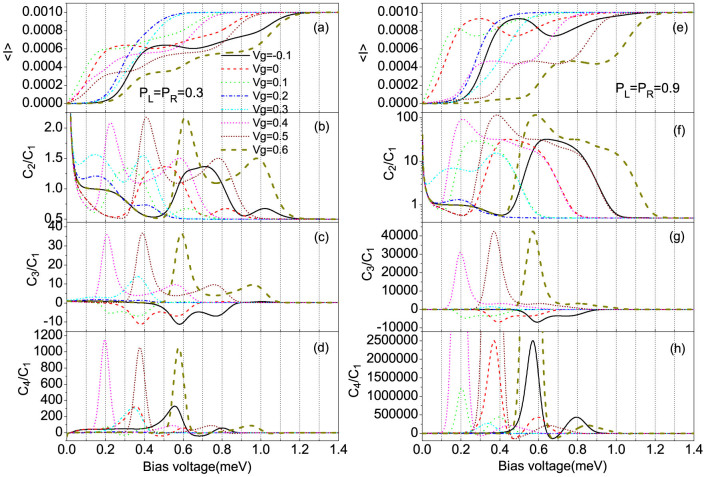
The average current (〈*I*〉), shot noise (*C*_2_/*C*_1_), skewness (*C*_3_/*C*_1_) and kurtosis (*C*_4_/*C*_1_) vs bias voltage for different gate voltages, here *C_k_* is the zero-frequency *k*-order cumulant of current fluctuations. (a), (b), (c) and (d) for *p_L_* = *p_R_* = 0.3; (e), (f), (g) and (h) for *p_L_* = *p_R_* = 0.9. The SMM parameters are the same as in Fig. 2.

We now consider the F-SMM-F system, the strengths of the spin-dependent SMM-electrode coupling are characterized by 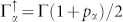 and 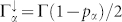, here we set *p_L_* = *p_R_* = *p*. For a small enough or negative gate voltage and relatively large spin polarization of the source and drain electrodes *p*, an obvious NDC is observed but weaker than that in the N-SMM-F system, especially for a large enough spin polarization *p*, see the solid and dashed lines in Figs. 4(a) and 6(a), and 4(e) and 6(e). While for a relatively large gate voltage, such as *V_g_* ≥ 0.4 meV, a weak NDC can be observed for a large enough spin polarization *p*, but that in the N-SMM-F system does not occur. Interestingly, for a small enough or negative gate voltage, the shot noise in the NDC region is dramatically enhanced and reaches up to a super-Poissonian value, see the solid and dashed lines in Figs. 6(b) and 6(f); whereas for a large enough gate voltage the formed super-Poissonian shot noise in the NDC region is decreased, see the short dashed, short dash-dotted and thick dashed lines in Fig. 6(f). This characteristic depends on the formation mechanism of the NDC, which is illustrated by the examples of *V_g_* = −0.1 and *V_g_* = 0.6 with *p* = 0.9.

**Figure 7:**
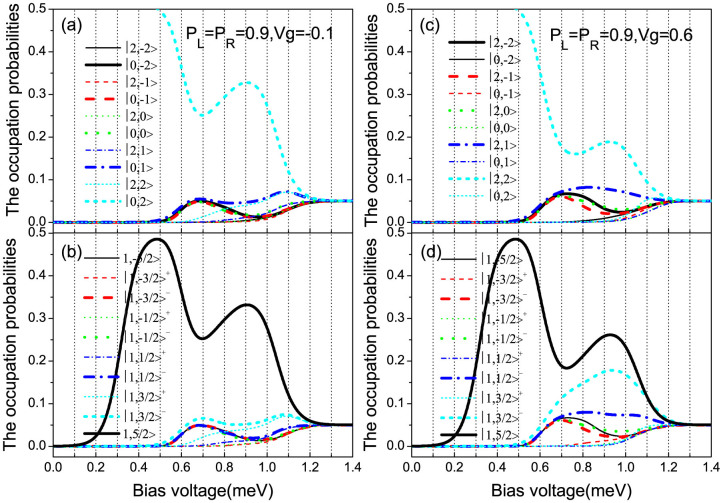
The occupation probabilities of the SMM eigenstates vs bias voltage for different gate voltages with *p_L_* = *p_R_* = 0.9. (a) and (b) for *V_g_* = −0.1; (c) and (d) for *V_g_* = 0.6. The SMM parameters are the same as in Fig. 2.

For a negative gate voltage *V_g_* = −0.1, the fast transport channel 
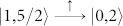
 first enters the bias voltage window. When the bias voltage increases up to about 0.48 meV, the new spin-up electron tunneling processes, namely, 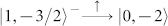, 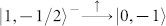, 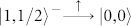, 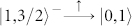, and the spin-dowm electron tunneling processes, namely, 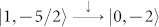, 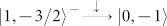, 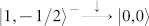, 
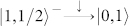
, 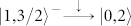 begin to participate in the quantum transport, see Figs. 7(a) and 7(b). This leads to the current magnitude of the fast transport channel 
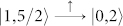
 decrease, but the increased current magnitudes of the new opened transport channels are too small to compensate the decreased current magnitude of 
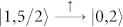
. Thus, a NDC region can form, in which the corresponding shot noise is rapidly enhanced by the active competition between the fast channel of current decreasing and the slow channels of current increasing, and reaches up to a large super-Poissonian value, see the solid line in Fig. 6(f). With further increasing the bias voltage, the formed active competition between the fast channel of current decreasing and the slow channels of current increasing is weakened and even disappears, but the effective competition between the spin-up and spin-down electron tunneling processes is still valid due to 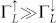 and 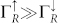, thus, the value of the formed super-Poissonian begins to continually decrease but still remains super-Poissonian distribution. When the bias voltage increases up to 0.8 meV, the current magnitudes of the transport channels originating from the transitions between the double- and singly-occupied eigenstates are already larger than that of the some transport channels originating from the transitions between the singly- and empty-occupied eigenstates, for example, 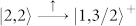. In this case, the formed effective competition between the fast and slow transport channels is suppressed and finally destroyed due to these transport channels via the transitions from the double- to singly-occupied eigenstates entering the bias voltage. Consequently, the super-Poissonian shot noise is decreased quickly up to a sub-Poissonian value, see the solid line in Fig. 6(f).

Compared with the *V_g_* = −0.1 case, for *V_g_* = 0.6 the transport channel 
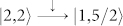
 first participates in the quantum transport. When the bias voltage increases up to about 0.48 meV, the spin-up transport channels 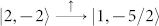, 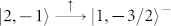, 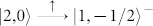, 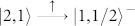, 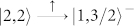, and the spin-down transport channels 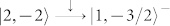, 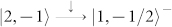, 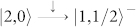, 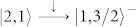 can be opened, while the current magnitude of the transport channel 
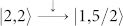
 begin to decrease, see Figs. 7(c) and 7(d). For the 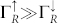 case, the decreased current magnitude of the spin-down transport channel 
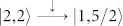
 is smaller than the increased current magnitudes of the new opened transport channels, thus, the NDC does not appear. Whereas the active competition between the fast channel of current decreasing and the slow channels of current increasing in a relatively small bias voltage range can form but soon be destroyed, so that the shot noise is significantly enhanced up to a very large super-Poissonian value, then this value begins to decrease but still remains super-Poissonian distribution due to the effective competition between the spin-up and spin-down electron tunneling processes being still valid, see the thick dashed line in Fig. 6(f). In particular, it is interesting note that the current magnitudes of the transport channels 
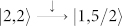
 and 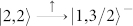 increase with further increasing the bias voltage, while the current magnitudes of the other transport channels 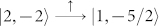, 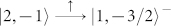, 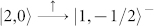, 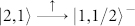, 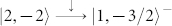, 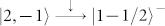, 
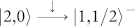
 and 
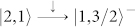
 decrease. This feature leads to the occurrence of a weak NDC. In this NDC bias voltage range, however, the super-Poissonian shot noise value continually decreases, see the thick dashed line in Fig. 6(f). When the transport channels originating from the transitions from the singly- to empty-occupied eigenstates enter the bias voltage, the physical mechanism of decreasing super-Poissonian shot noise is the same as the *V_g_* = −0.1 case, namely, the formed effective competition between the spin-up and spin-down electron tunneling processes is weakened even destroyed by these current increased transport channels. This is responsible for the super-Poissonian shot noise being decreased to a sub-Poissonian value.

We now study the skewness and kurtosis properties of the transport current in the super-Poissonian shot noise bias voltage regions. It is well known that the skewness and kurtosis (both its magnitude and sign) characterize, respectively, the asymmetry of and the peakedness of the probability distribution around the average transferred-electron number 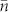 during a time interval t, thus that provide further information for the counting statistics beyond the shot noise. In the N-SMM-F system with a given small enough or negative gate voltage, for a relatively large *p_R_*, the skewness shows a crossover from a large negative to a relatively small positive values, while the kurtosis shows a crossover from a large positive to a relatively small negative values, see the solid and dashed lines in Figs. 4(c) and 4(d); whereas for a large enough *p_R_*, the transition of the skewness from a large negative to a large positive values takes place and forms a Fano-like resonance, see the solid, dashed and dotted lines in Fig. 4(g), while the transitions of the kurtosis from a large positive to a large negative values and then from the large negative to a large positive values take place, and form the double Fano-like resonances, see the solid, dashed and dotted lines in Fig. 4(h). In contrast with a small enough or negative gate voltage, for a large enough gate voltage, the skewness and kurtosis for a relatively large *p_R_* show, respectively, the crossovers from a large positive to a relatively small negative values and from a small positive to a relatively large negative values, see the short dash-dotted and thick dashed lines in Figs. 4(c) and 4(d); while for a large enough *p_R_*, the skewness and kurtosis show, respectively, the crossovers from a small positive to a relatively large negative values and from a small negative large to a relatively large positive values, see the short dash-dotted and thick dashed lines in Figs. 4(g) and 4(h), but the variations in the magnitudes of the skewness and kurtosis are much smaller than that for a small enough or negative gate voltage, see Figs. 4(g) and 4(h). As for the F-SMM-F system with a given relatively large *p*, the skewness for a small enough or negative gate voltage shows a large negative value, see the solid, dashed and dotted lines in Figs. 6(c) and 6(g), whereas for a large enough gate voltage that shows a large positive value, see the short dashed, short dash-dotted and thick dashed lines in Figs. 6(c) and 6(g); while the kurtosis shows the double-crossover from a large positive to a relatively small negative values and then from the negative to a large positive values but the latter has a remarkable variation in the magnitude of the kurtosis, see Figs. 6(d) and 6(h). Moreover, we found that the magnitudes of the skewness and kurtosis are more sensitive to the active competition between the fast channels of current decreasing and the corresponding slow channels of current increasing than the shot noise, see the short dashed, short dash-dotted and thick dashed lines in Figs. 2, 4 and 6.

## Discussion

Now, we discuss the feasibility of the experimental tests of the current noise properties of the SMM junctions. The differential conductance of the Mn_12_^1^,^2^,^8^, Fe_4_^5^,^10^ and TbPc_2_^7^,^9^,^11^ SMM junctions has been experimentally demonstrated. On the other hand, the high-order current cumulants of electron transport through a semiconductor quantum dot have been realized experimentally^51^,^52^, especially the fifteen-order cumulants can be extracted from the high-quality real-time single-electron measurements^52^. Consequently, in principle, the quantum transport of individual electrons through a SMM can be detected using a quantum point contact^51^,^52^ or a carbon nanotube field-effect transistor^28^ acting as a charge sensor. This provides a possible opportunity to experimentally test the predicted negative differential conductance, super-Poissonian shot noise and the high-order current cumulants of the SMM junctions considered here. Moreover, it should be noted that the shot noise in a SMM junction depends on the internal level structure of the SMM^32^,^33^,^34^, the left-right asymmetry of the SMM-electrode coupling^32^,^33^,^34^ and the spin polarization of the source and drain electrodes. However, in the present SMM junctions fabricated by the break-junction and electromigration techniques^1^,^2^,^5^,^10^, the angle of the easy axis of the SMM with respect to the polarization vectors of the source and drain electrodes^5^,^10^, and the left-right asymmetry of the SMM-electrode coupling varies from sample to sample. Thus, the shot noise properties of the SMM junction varies from sample to sample.

In summary, we have studied the the NDC and super-Poissonian shot noise properties of electron transport through a SMM weakly coupled to two electrodes with either one or both of them being ferromagnetic, and analyzed the skewness and kurtosis properties of the transport current in the super-Poissonian shot noise regions. It is demonstrated that the occurrences of the NDC and super-Poissonian shot noise depend sensitively on the spin polarization of the soure and drain electrodes and the applied gate voltage. For the F-SMM-N system, when the transition from the double- to singly-occupied eigenstates first enters the bias voltage window, which corresponds to a large enough gate voltage, the super-Poissonian shot noise is observed for a large enough spin polarization of left electrode *p_L_*. As for the N-SMM-F system, the NDC and super-Poissonian shot noise can be observed for a relatively large spin polarization of right electrode *p_R_* and a small enough or negative gate volatge, especially for a large enough *p_R_* a strong NDC and a very large value of the super-Poissonian shot noise appear, and the shot noise in the NDC region is first enhanced up to a super-Poissonian value and then is decreased but still remains super-Poissonian distribution for a large enough *p_R_*; while for a large enough gate voltage and a relatively large *p_R_* the super-Poissonian shot noise is only observed. Compared with the N-SMM-F system, for the F-SMM-F system a relatively weak NDC and a large super-Poissonian shot noise bias voltage range are observed; whereas the formed super-Poissonian shot noise in the NDC region is continually decreased for a large enough gate voltage and spin polarization of left and right electrodes *p*. Furthermore, the transitions of the skewness and kurtosis from a large positive (negative) to a large negative (positive) values are also observed, which can provide a deeper and better understanding of electron transport through single-molecule magnet junctions. The observed NDC and super-Poissonian shot noise in the SMM system can be qualitatively attributed to the effective competition between the fast and slow transport channels, and the NDC properties suggest a gate-voltage-controlled NDC molecular device.

## Methods

The SMM-electrode coupling is assumed to be sufficiently weak, so that the sequential tunneling is dominant. The transitions are well described by the quantum master equation of a reduced density matrix spanned by the eigenstates of the SMM. Under the second order Born approximation and Markov approximation, the particle-number-resolved quantum master equation for the reduced density matrix is given by^53^,^54^
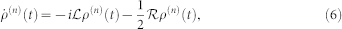
with
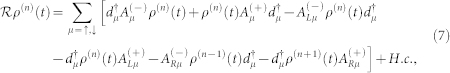
where 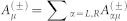, 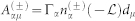, 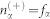 and 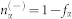 (*f_α_* is the Fermi function of the electrode *α*). Liouvillian superoperator 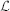 is defined as 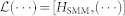. *ρ*^(*n*)^ (*t*) is the reduced density matrix of the SMM conditioned by the electron numbers arriving at the right electrode up to time *t*. In order to calculate the first four cumulants, one can define 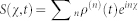. According to the definition of the cumulant generating function 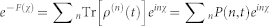, we evidently have *e*^−*F*(*χ*)^ = Tr[*S*(*χ*, *t*)], where the trace is over the eigenstates of the SMM. Since Eq. (6) has the following form
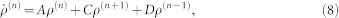
*S* (*χ*, *t*) satisfies
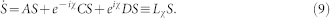
In the low frequency limit, the counting time is much longer than the time of electron tunneling through the SMM. In this case, *F* (*χ*) can be expressed as^55^,^56^,^57^,^58^
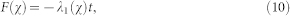
where *λ*_1_ (*χ*) is the eigenvalue of *L_χ_* which goes to zero for *χ* → 0. According to the definition of the cumulants, one can express *λ*_1_ (*χ*) as
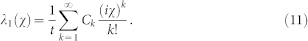
Here, the first four cumulants *C_k_* are directly related to the transport characteristics. For example, the first-order cumulant (the peak position of the distribution of transferred-electron number) 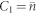 gives the average current 〈*I*〉 = *eC*_1_/*t*. The zero-frequency shot noise is related to the second-order cumulant (the peak-width of the distribution) 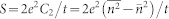. The third-order cumulant 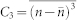 and four-order cumulant 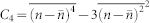 characterize, respectively, the skewness and kurtosis of the distribution. Here, 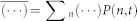. In general, the shot noise, skewness and kurtosis are represented by the Fano factors *F*_2_ = *C*_2_/*C*_1_, *F*_3_ = *C*_3_/*C*_1_ and *F*_4_ = *C*_4_/*C*_1_, respectively. Moreover, the Fano factors *F*_2_ < 1, *F*_2_ = 1 and *F*_2_ > 1 describe, respectively, the sub-Poissonian shot noise, the Poisson noise and the super-Poissonian shot noise.

The low order cumulants can be calculated by the Rayleigh–Schrödinger perturbation theory in the counting parameter *χ*, which was developed in Refs. 57, 59, 60. In order to calculate the first four current cumulants we expand *L_χ_* to four order in *χ*
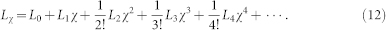
Along the lines of Refs. 57, 59, 60, we define the two projectors 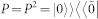 and *Q* = *Q*^2^ = 1 − *P*, obeying the relations *PL*_0_ = *L*_0_*P* = 0 and *QL*_0_ = *L*_0_*Q* = *L*_0_. Here, |0〉〉 being the steady state *ρ^stat^* is the right eigenvector of *L*_0_, namely, *L*_0_|0〉〉 = 0, and 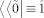 is the corresponding left eigenvector. In view of *L*_0_ being singular, we also introduce the pseudoinverse according to 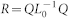, which is well-defined due to the inversion being performed only in the subspace spanned by *Q*. After a careful calculation, *λ*_1_ (*χ*) is given by
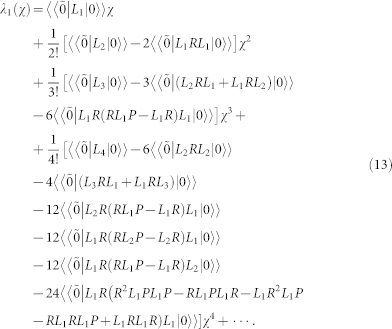
From Eqs. (11) and (13) we can identify the first four current cumulants:
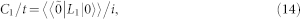

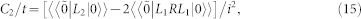

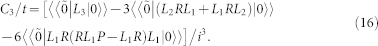

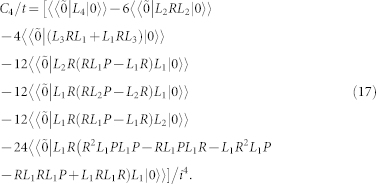


The above four equations are the starting point of the numerical calculation.

## Author Contributions

H.B.X. conceived the idea and designed the research and performed calculations. J.Q.L. and W.M.L. contributed to the analysis and interpretation of the results and prepared the manuscript.
